# The Healing Effect of *Plantago Major* and *Aloe Vera* Mixture in Excisional Full Thickness Skin Wounds: Stereological Study

**DOI:** 10.29252/wjps.8.1.51.

**Published:** 2019-01

**Authors:** Soheil Ashkani-Esfahani, Mahsima Khoshneviszadeh, Ali Noorafshan, Ramin Miri, Shima Rafiee, Kimia Hemyari, Sina Kardeh, Omid Koohi Hosseinabadi, Dorna Fani, Elnaz Faridi

**Affiliations:** 1 Student Research Committee, Shiraz university of Medical Sciences, Shiraz, Iran;; 2 Medicinal and Natural products Chemistry Research Center, Shiraz university of Medical Sciences, Shiraz, Iran;; 3 Histomorphometry and Stereology Research Center, Shiraz University of Medical Sciences, Shiraz, Iran;; 4 Student Research Committee, International branch, Shiraz University of Medical Sciences, Shiraz, Iran;; 5 Burn and Wound Healing Research Center, Division of Plastic and Reconstructive Surgery, Department of Surgery, Shiraz University of Medical Sciences, Shiraz, Iran;; 6 Center of Comparative and Experimental Medicine, Shiraz University of Medical Sciences, Shiraz, Iran

**Keywords:** Wound healing, Fibroblast, Collagen, Vascularization, Plantago major, Aloe vera, Stereology, Rat

## Abstract

**BACKGROUND:**

Previous studies indicated that both *Plantago major* and *Aloe vera* have anti-inflammatory, tissue regeneration, antioxidant, and immune-stimulatory effects. It is assumed that a mixture of these two herbal medicines may provide a potent material in treatment of skin wound injuries. Therefore, in this study we investigated the effects of *Plantago major* and *Aloe vera* mixture in the process of wound healing in rat models according to stereological parameters.

**METHODS:**

In an experiential study, 36 male Sprague-Dawley rats (200±20 g) were randomly assigned into three groups (n=12): The control group which received no treatment, gel base treated group, and the 5% *Plantago major* and 5% *Aloe vera* mixture gel treated group (PA group). Treatments were done every 24 hrs for 15 days. Wound closure rate, volume densities of the collagen bundles and the vessels, vessel’s length density and mean diameter, and fibroblast populations were estimated using stereological methods.

**RESULTS:**

PA treated group showed faster wound closure rate in comparison with control and gel-base groups (*p*<0.05). Numerical density of fibroblasts, volume density of collagen bundles, mean diameter, and volume densities of the vessels in PA group were significantly higher than the control and the gel-base treated groups (*p*<0.05).

**CONCLUSION:**

We showed that *Plantago major* and *Aloe vera* mixture has the ability to improve wound healing by enhancing fibroblast proliferation, collagen bundle synthesis and re-vascularization in skin injuries.

## INTRODUCTION

Wound healing as a dynamic and normal biological process in body is consisted of four programmed and overlapping phases of hemostasis, inflammation, proliferation, and remodeling.^1^^-^^3^ This process involves fibroblast activation and migration, re-epithelization, proliferation of endothelial cells, and angiogenesis in the damaged area.^4^^,^^5^ Immediately after the injury, the innate immune system is activated; wound repair and regeneration process begins by interactions between growth factors and extracellular matrix, cytokines and etc.^6^^,^^7^ Also, it was shown that inflammatory response and oxidative reactions play important roles in this essential process.^2^^,^^4^

*Plantago major* leaves from *Plantagiaceae* family have been used as a wound healing herbal agent for many years in traditional medicine.^8^ Some components like polysaccharides, lipids, caffeic acid derivatives, flavonoids, iridoid glycosides, and terpenoids exist in this plant extract which have anti-inflammatory, antioxidant, analgesic, immune-modulatory, anti-ulceration, and weak antibiotic activities.^9^^,^^10^ On the other hand, *Aloe vera* from *Liliaceae* family is another herbal medicine which is also used in the modern world due to its pharmacological actions including wound healing, antioxidant, anti-scar formation, anti-inflammatory, re-epithelialization, and immune-stimulatory effects.^11^^,^^12^

Also, it was reported that *Aloe vera* increased the collagen content of the granulation tissue as well as its degree of cross linking as seen by increasing aldehyde content and decreased acid solubility.^13^^,^^14^ According to previous studies on different impacts of both *Plantago major* and *Aloe vera*, some of which are supposed to influence the process of wound healing, it is assumed that a mixture of these two herbal medicines may provide a potent material in treatment of skin wounds. Thus, in this study, we aimed to determine the healing effects of this mixture on full-thickness skin wounds in rat models by using histomorphometrical and stereological parameters. 

## MATERIALS AND METHODS

The fresh leaves of plants were collected from rural area of Bushehr city, Iran. The taxonomic identity of the plant was confirmed by Dr. R. Miri, and voucher specimen (*Aloe vera* No: 2029, *Plantago major* No: 3491) was deposited at Botany Department Herbarium, Shiraz University of Medical Sciences, Shiraz, Iran. The plant materials were washed under running tap water to remove the surface pollutants and the different parts of leaves were separated mechanically. The separated parts were air dried under shade. The dried sample was powdered and used for further studies.

The powdered leaves materials were packed in small thimbles separately and extracted successively with organic solvents such as petroleum ether, chloroform and acetone in the increasing order of polarity using soxhlet apparatus. Each time before extracting with the next solvent, the thimble was air dried. The different solvent extracts were concentrated by rotary vacuum evaporator (Yamato RE300, Japan) and then air dried. The dried extract obtained with each solvent was weighed. The percentage of yield was calculated in terms of the air dried weight plant material (1 mg/ml of respective organic solvents), the extract obtained was used for the assessment of further investigation.

Hydroalcoholic extracts of the herbs were prepared and mixed by 1:1 ratio. In order to facilitate the application of the extract, we prepared 10% mixture gel (PA) by dissolving 5 mg of *Aloe vera* plus 5 mg of *Plantago major* in 2 mL of distilled water, and then transferring the solution into 2% carboxymethyl cellulose (CMC) (2 g CMC dissolved in 98 mL of distilled water). The base of gel was also supplied by the same method, but without the herbal component.

Thirty six healthy male Sprague-Dawley rats weighing 200±20 g were housed between 2 to 3 months age under standard environmental condition. The animals kept in standard cages with standard pellet diet and water *ad libitum *in animal laboratory of Shiraz University of Medical Sciences. These animals were randomly assigned into three groups (n=12). One group was treated with vehicle gel (Gel base) as a placebo treated group. The second group was treated with 10% mixture of *Aloe vera* and *Plantago major*, and the third wounded group labeled as the control group which received no treatment. 

This study was blinded to reduce the risk of bias. On the day 0, under general anesthesia, a ~1 cm^2^ circular full-thickness wound was made on the posterior surface of each rat’s neck. The topical administrations were done in a standard manner just after the wounding and were repeated every 24 h until the last day of the study (the day in which at least one wound in any groups of rats was entirely closed). At the end, the animals were sacrificed with a high dose of ether. A full-thickness circular skin sample with a 1 cm margin around the wound area was removed from the wound’s site and fixed in buffered formaldehyde for stereological evaluation. All animal experiments in this study protocol were approved by the Animal Ethics Committee of the Shiraz University of Medical Sciences and the animal care was in accordance with their moral guidelines.

**Fig. 1:**
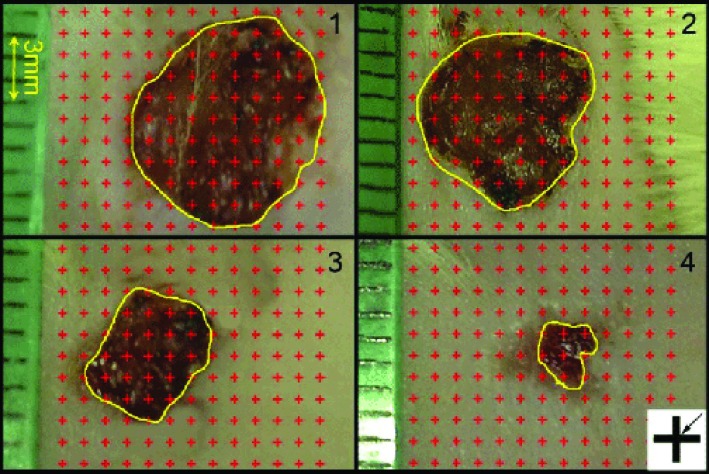
Digital photographs were captured from the wound surfaces every four day to measure the wound area. The total number of points within the wound borders (yellow line) was counted. At the corner of this figure, a cross is presented. The right upper corner of the cross is considered as the point (arrow), and it is counted only if the right upper corner hits the wound surface

To determine the rate of wound area reduction, digital photographs were captured from the wound surfaces every other day. A standard ruler was laid at the wound level to find the magnification on the computer monitor (Figure 1). The wound area was calculated by using a method introduced by Noorafshan *et al.*^15^ Nine pieces of the skin samples, each about 1 mm^2^, were cut and prepared for stereological analysis, in a systematic randomized sampling. Parts were embedded in a cylindrical paraffin block. Isotropic uniformly random (IUR) sections of the blocks with 5 μm and 15 μm width were obtained^16^ and blemished with both Hedenhain’s azan and hematoxylin-eosin stains. 

**Fig. 2:**
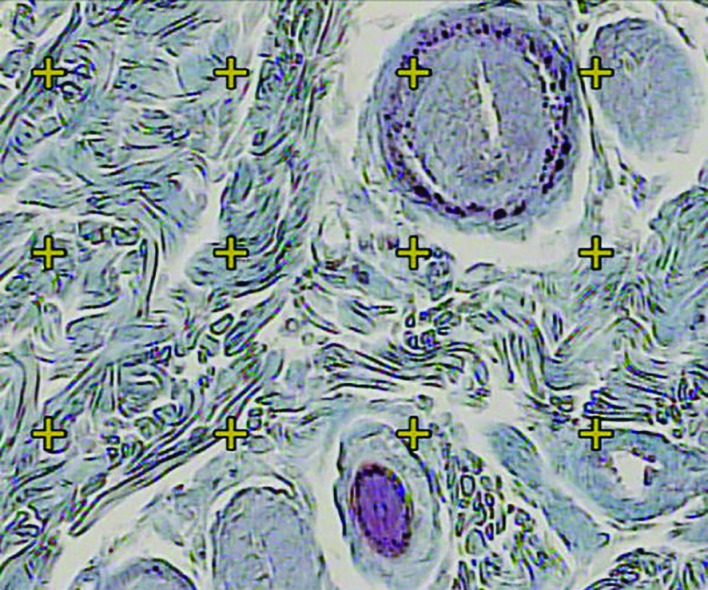
Using a grid of points on dermis, the volume density (Vv (collagen/dermis)) of the collagen fibers was estimated. The total number of the points hitting the bundles is divided by the total number of the points hitting dermis. A cross is counted only if the right upper corner hits the tissue (Hedenhain’s azan stain) (×450)

**Fig. 3:**
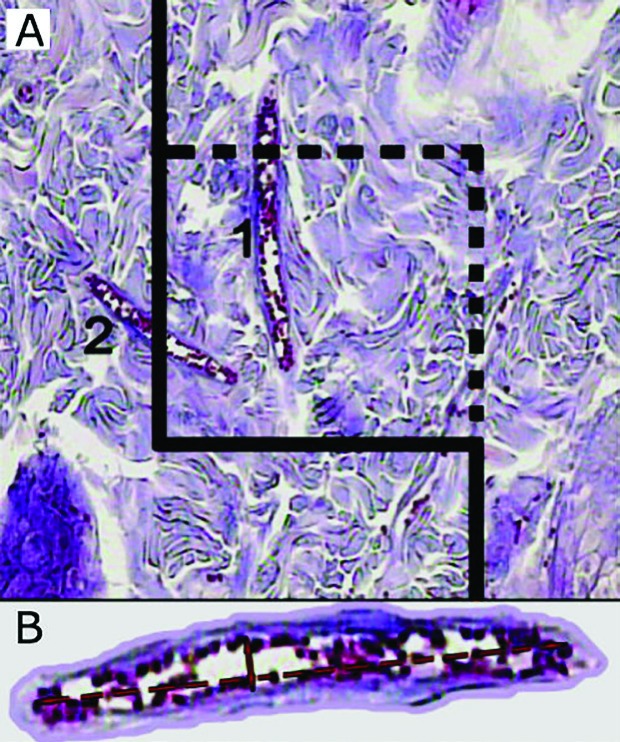
A) To estimate the vessel’s Lv and mean diameter, a counting frame is randomly laid on the wound dermis at magnification of 450. Any vessel which lies in this frame or touches the dotted borders is selected and vessels which touch the continuous lines are omitted. B) The short axis of the vessel is used to measure the mean diameter of the vessel (Hedenhain’s azan stain).

**Fig. 4:**
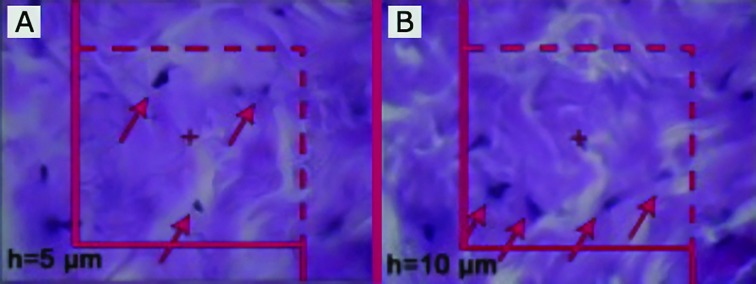
A) counting frame is used to estimate the numerical density (Nv) of the fibroblasts (The nuclei are unclear at the first 5 µm optical section). B) Any nucleus in the counting frame or touching the dotted lines and not the continuous lines, which comes into the maximal focus within the next traveling 5 µm optical section, is counted (Hedenhain’s azan stain ×2000).

Microscopic analyses of the dermis were done by using a video-microscopy system made up of a microscope (E-200; Nikon™; Japan) and a video camera and a flat monitor. The volume densities of the collagen bundles, vessels, and hair follicles, were estimated by using unbiased stereological methods (Figure 2). The length density (Lv) and the mean diameters of the vessels were estimated at final magnification of X450 by employing the 5 μm thickness slides for stereological analysis and by using a reported method (Figure 3). The numerical density (Nv; number of the cells per unit volume of the dermis) of the fibroblasts was approximated by employing the 15 μm slides and the “optical dissector” method (Figure 4).^17^ The data were accumulated, analyzed, and informed as mean and standard deviation (mean±SD). In addition, the statistical comparisons between the groups were executed by the SPSS statistical software (version 16.0, Chicago, IL, USA). Mann-Whitney U-test was used in order to analyze the data. Moreover, P≤0.05 was supposed as statistically significant.

## RESULTS

**Fig. 5:**
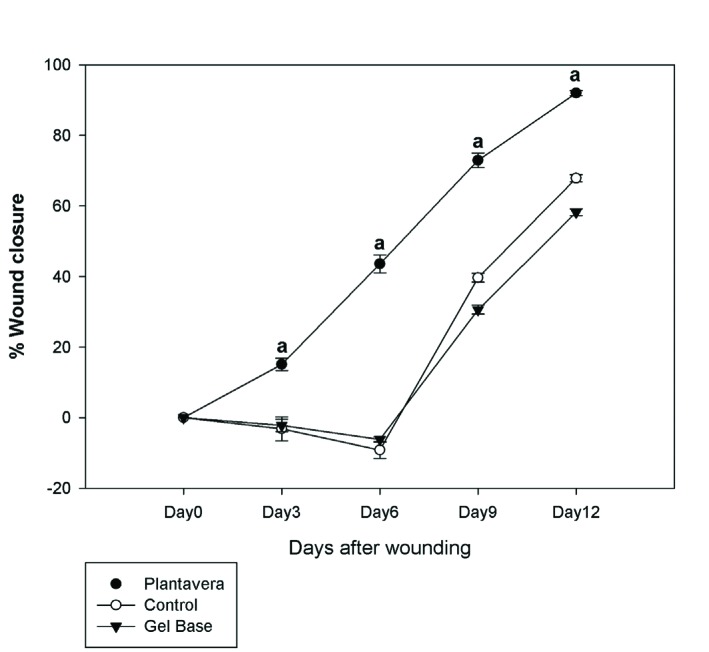
Wound closure rates in the control, PA treated, and gel-base treated groups. Each point represents mean±SD of the wounds of each group. The wound closure rate had significantly increased in PA treated group compared to the control and gel-base groups (*p*<0.05)

As it was shown in Figure 5, wound closure rate of control and gel-base groups were approximately similar. Based on the results of this study, PA treated group (7.67% closure per day average) showed faster wound closure rate in comparison with control (5.65%/day) and gel-base (4.85%/day) groups since the beginning of the measurements (*p*<0.05). Numerical density of fibroblasts in the dermis of the PA group was higher than that of the control and gel-base groups. As it is shown in Table 1, numerical density of the fibroblasts in PA group was reported 1.03 time higher than the control group (*p*<0.001) and 1.91 time higher than the gel-base group (*p*<0.001). 

**Table 1:** Mean (SD) of the numerical density of the fibroblasts (×10^3^ per mm^3^), volume densities of the collagen bundles (Vv_collagen/dermis_; %) and vessels (Vv_vessel/dermis_; %), length density (mm/mm^3^) and mean diameter (µm) of vessels in the dermis of the wounded rats treated with PA gel , gel-base and untreated wounded group (Control)

**Groups **	**Fibroblast**	**Collagen**	**Vessel**	**Length density**	**Mean diameter **
	**Numerical density**	**Volume density **	**Volume density **		
Gel-base	17.33 (8.28)	55% (5%)	3.25% (3%)	20.21(8.10)	2.21 (0.44)
PA	37.91* (6.45)	79% (7%)†	7.00% (4%)*	20.08 (5.50)	3.03 (2.15)†
Control	18.02 (6.66)	49% (5%)	2.40% (3%)	17.16 (7.26)	1.22 (.28)

* *p*<0.05, PA group vs. control and gel-base group.

† *p*<0.05, PA group vs. control group.* Plantago major* and *Aloe ver*a (PA).

The mean of volume density of the collagen bundles was 79±7% in Plantavera group which was 49% and 55% higher than control and the gel-base groups (*p*<0.005), respectively. Vessel’s volume density was 7.00±4.05% in PA group which was considered significant in comparison to the control and the gel-base groups (*p*<0.05). Length density of the vessels was 20.08% in PA group that had no significant difference with the control and the gel-base groups. Also, as it is shown in Table 1, in comparison to the PA treated group, the mean of the vessel diameters was 37% lower in the gel-base group (*p*=0.321) and 148% lower in the control group (*p*=0.048; Table 1).

## DISCUSSION

It is believed that dermal reconstruction depends on cell proliferation, extracellular matrix deposition, wound contracture, and angiogenesis.^18^^,^^19^ It had been revealed that tissue regeneration was highly dependent on inflammatory responses and oxidative reactions.^20^^,^^21^ Evidences revealed that both *Plantago major* and *Aloe vera* have antioxidant and anti–inflammatory activity and can improve collagen and proliferation of fibroblast.^22^^,^^23^ Zubari and his colleagues showed that an aqueous extract of PA leaves stimulated wound healing in a rat model.^24^

In another study conducted by Amini *et al.*, methanol and aqueous extracts of *Plantago major* leaves showed stimulating effects on burn wound healing in rat.^25^ Also leaf extracts of *Plantago major* indicated to enhance cell proliferation and migration in vitro which are crucial parts of the wound healing process.^26^ A study is conducted by Eshghi *et al.* to show the effects of *Aloe vera* cream in reducing postoperative pain and its promotion of wound healing after open hemorrhoidectomy. Their results showed that this cream significantly improved the wound healing after hemorrhoidectomy and the anti-inflammatory effects contribute to relief of postoperative pain.^27^

Also effects of *Aloe vera* on burn wound healing were shown in a study conducted in guinea pig. The study reported that the gel extract of *Aloe vera* increases the healing rate, and reestablished the vascularity of burn tissues. They reported that these effects might be due to several mechanisms including an increasing collagen synthesis and rate of epithelialization by the effect of Acemanan (mannose-6 phosphate).^28^^,^^29^

The results of our study indicated that the mixture of *Plantago major* and *Aloe vera* (PA) improved fibroblast proliferation, collagen bundle synthesis and re-vascularization in skin injuries. Considering the findings of the present study and previous reports on the effects of PA mixture, this compound is assumed to have the ability to be introduced as a promising treatment for full thickness skin wounds; However, further studies are required to determine the alterations induced by this mixture in intercellular and extracellular signaling pathways leading to improvement of wound healing followed by clinical studies which are also needed to be conducted in order to evaluate the influence of this two substances on human skin wounds. We showed that *Plantago major* together with *Aloe vera* has the ability to improve wound healing by enhancing fibroblast proliferation, collagen bundle synthesis and revascularization in skin injuries.
